# The benzoyl-CoA pathway serves as a genomic marker to identify the oxygen requirements in the degradation of aromatic hydrocarbons

**DOI:** 10.3389/fmicb.2023.1308626

**Published:** 2024-01-09

**Authors:** Camila Monserrat Godínez-Pérez, Antonio Loza, Juan Manuel Hurtado, Rosa-María Gutiérrez-Ríos

**Affiliations:** Departamento de Microbiología Molecular, Instituto de Biotecnología, Universidad Nacional Autónoma de México, Cuernavaca, Morelos, Mexico

**Keywords:** monoaromatic hydrocarbons, benzoate-CoA ligase, genomic markers, multipartite chromosome, aerobic and anaerobic degradation pathways

## Abstract

The first step of anaerobic benzoate degradation is the formation of benzoyl-coenzyme A by benzoate-coenzyme A ligase (BCL). The anaerobic route is steered by benzoyl-CoA reductase, which promotes benzoyl-CoA breakdown, which is subsequently oxidized. In certain bacteria at low oxygen conditions, the aerobic metabolism of monoaromatic hydrocarbons occurs through the degradation Box pathway. These pathways have undergone experimental scrutiny in Alphaproteobacteria and Betaproteobacteria and have also been explored bioinformatically in representative Betaproteobacteria. However, there is a gap in our knowledge regarding the distribution of the benzoyl-CoA pathway and the evolutionary forces propelling its adaptation beyond that of representative bacteria. To address these questions, we used bioinformatic procedures to identify the BCLs and the lower pathways that transform benzoyl-CoA. These procedures included the identification of conserved motifs. As a result, we identified two motifs exclusive to BCLs, describing some of the catalytic properties of this enzyme. These motifs helped to discern BCLs from other aryl-CoA ligases effectively. The predicted BCLs and the enzymes of lower pathways were used as genomic markers for identifying aerobic, anaerobic, or hybrid catabolism, which we found widely distributed in Betaproteobacteria. Despite these enhancements, our approach failed to distinguish orthologs from a small cluster of paralogs exhibiting all the specified features to predict an ortholog. Nonetheless, the conducted phylogenetic analysis and the properties identified in the genomic context aided in formulating hypotheses about how this redundancy contributes to refining the catabolic strategy employed by these bacteria to degrade the substrates.

## Introduction

Aromatic compounds, found as lignin components, flavonoids, quinones, aromatic amino acids, or constituents of fossil fuels, are among the most widely distributed classes of organic compounds in nature (Fuchs et al., 2011; Díaz et al., 2013; Boll et al., 2020). Among these contaminants, those derived from fossil fuels and xenobiotic chemicals are recognized as contaminants that can enter the environment due to human activities (Seo et al., 2009; Prince et al., 2010; Fuchs et al., 2011). The greatest concern within these compound groups centers on their toxicity, prompting the investigation of efficient degradation techniques, where microorganisms play a crucial role because of their ability to use them as carbon and energy sources. Within the natural environment, the breakdown of aromatic substances is primarily managed by aerobic and anaerobic bacteria, as well as aerobic fungi, where bacteria are instrumental in carbon recycling from aromatic rings (Boll et al., 2020).

In the case of monoaromatic hydrocarbons, aerobic microorganisms utilize monooxygenase or dioxygenase enzymes to incorporate single or double atoms of molecular oxygen (O_2_) into substrates. This process leads to ring hydroxylation and cleavage (George and Hay, 2011). Moreover, microorganisms in oxygen-depleted environments can degrade monocyclic aromatic compounds (MACs) using various terminal electron acceptors, such as ferric, nitrate, sulfate, chlorate, perchlorate, or organic acids such as fumarate. These electron acceptors facilitate degradation coupled with respiration under anoxic conditions (Carmona et al., 2009).

The benzoyl-CoA pathway is widely distributed in nature since it catabolizes a wide range of compounds, such as benzene, toluene, ethylbenzene, and xylene (BTEX), as well as phenol, benzoates, phthalates, phenylpropane-derived compounds, phenylacetic acid, and phenylalanine (Harwood et al., 1998). Among the mentioned MACs, benzoate is commonly degraded by anaerobic bacteria. As a result, the processes involved in its catabolism have been studied in greater detail than those of any other anaerobic degradation pathway of aromatic compounds (Gibson and Harwood, 2002). Consequently, benzoate is considered a model compound for studying the central degradation pathway of aromatic compounds via benzoyl-CoA in anaerobic organisms, in which the first step of anaerobic benzoate degradation is its activation of benzoyl-CoA by benzoate-CoA ligase.

The BCL enzymes belong to the Class I superfamily of adenylate-forming enzymes (ANL superfamily) and subclass Ib, which include acyl and aryl coenzyme A (CoA) synthetases/ligases (Schmelz and Naismith, 2009; Clark et al., 2018; Arnold et al., 2021). Using ATP, it catalyzes the formation of a thioester bond between aromatic compounds and coenzyme A (CoA, CoASH). In the first step of this process, negatively charged benzoate oxygen attacks α-phosphate, the most positive phosphate in ATP, forming an aryl-adenylate intermediate and releasing pyrophosphate (PPi). Facultative aerobic organisms such as denitrifying and phototrophic bacteria use hybrid metabolism at low oxygen concentrations. These bacteria use oxygen to introduce hydroxyl groups, as in the classical aerobic pathway (Díaz et al., 2013), where these bacteria can reduce the aromatic ring and use CoA thioesters, as in anaerobic metabolism. In the hybrid aerobic pathway, the activation of benzoate to benzoyl CoA requires the action of BCL (Valderrama et al., 2012), and the enzymes used in the lower pathways are encoded by *box* genes.

The benzoyl-CoA pathway has been experimentally studied in some facultative anaerobes within Betaproteobacteria, such as *Thauera aromatica* (*T. aromatica*), A*romatoleum aromaticum* EbN1 (*A. aromaticum* EbN1), and *Azoarcus* spp. (Carmona et al., 2009), which were isolated from soils and sludges (Braun and Gibson, 1984; Sun et al., 2014); oil sand tailing ponds (Golby et al., 2012); wastewater treatment systems from petroleum refineries (Silva et al., 2012); coke oven wastewater (Sueoka et al., 2009); and wastewater treatment plants (Thomsen et al., 2007; Rabus et al., 2014); and the photosynthetic bacterium *Rhodopseudomonas palustris* CGA009 (*R. palustris* CGA009), an Alphaproteobacteria member isolated from swine waste lagoons, earthworm droppings, coastal marine sediments, and pond water (Larimer et al., 2004).

The genomes of *T. aromatica, A. aromaticum* EbN1, and *Azoarcus* spp. present two functional copies of the BCL in the chromosome, colocalizing with *box* gene clusters or the anaerobic benzoate metabolism gene cluster (Valderrama et al., 2012; Suvorova and Gelfand, 2019). On the other hand, *Burkholderia xenovorans* LB400, reclassified as *Paraburkholderia xenovorans* LB400 (*P. xenovorans* LB400), displays a different genomic arrangement. It encodes two copies of the BCL and *box* genes pathway, one located in the main chromosome and the other in a secondary replicon (megaplasmid) (Denef et al., 2005). However, the presence of these paralogous copies remains to be elucidated. An analysis at the proteomic level showed that the differential regulation of the pathways located on the main chromosome and in the megaplasmid was related to the cell growth phase and the substrates added (Denef et al., 2005). Another study hypothesized that the catalytic efficiency of BCLs would differ for specific substrates (Bains and Boulanger, 2007). Despite these efforts, there is still no answer to this question. Therefore, one of the aims of this work will be to try to respond to this interrogant.

Thousands of microbial genomes collected from distinct environments are available, which provides an enormous opportunity to extend our knowledge of the mechanisms underlying hydrocarbon degradation. In this vein, a recent study developed 37 dedicated matrices to identify gene markers to predict the hydrocarbon degradation potential held by microbial genomes and metagenomes (Khot et al., 2022). Nevertheless, identifying a single enzyme as a genomic marker does not reveal the probable mechanisms involved in the biodegradation of a substrate. In this context, an investigation dedicated to identifying members of the ANL family revealed more than 50,000 potential biosynthesis-related gene clusters within bacterial, fungal, and plant genomes. Machine learning techniques were used to classify ANLs that were identified using the extensively conserved AMP-binding domain (PF00501) (Robinson et al., 2020). The findings in this study showed, that aryl enzymes were more closely related to different protein subfamilies than to each other, indicating that computational methods for identifying and classifying ANLs can still be improved. Hence, this study integrates various bioinformatic methodologies to improve the identification of ANLs by employing BCL and its downstream pathways as a model, which we suggest as potential genomic markers. As a final step of recognition, we introduced novel matrices founded on ungapped motifs, which correlate with catalytic and substrate recognition properties specific to BCLs. These findings demonstrated the importance of these matrices in accurately distinguishing BCLs from other aryl-CoA ligases.

As a result, we found that the predicted BCLs are widespread in Betaproteobacteria, less conserved in Alphaproteobacteria, barely distributed in Gamma- and Deltaproteobacteria, and absent from these taxonomic classes. The prediction of orthologs of the lower pathways was essential for predicting the potential of these species to catabolize benzoyl-CoA anaerobically and aerobically, revealing that the metabolic context should be used to define metabolic markers. While our approach significantly enhanced ortholog searches, it encountered challenges in handling a subset of species within the Rhodocyclales and Burkholderiaceae families. This subset exhibited two copies of BCLs sharing all the proposed features, posing difficulties in determining the ortholog. Upon closer examination of the genomes, it became evident that in a specific subset of Burkholderiaceae, one of the copies was encoded on a secondary chromosome. Therefore, we generated a phylogenetic tree to comprehend the evolutionary forces driving the preservation of these paralog copies and the meaning of their placement in the chromosome or megaplamids. The tree revealed that the copies in chromids or megaplasmids formed a distinct clade far from the root, indicating their acquisition during a subsequent evolutionary event. Our analysis also revealed that these copies continue to undergo selective pressures that guarantee their conservation, providing the bacteria with several strategies for utilizing specific MACs.

## Methods

### Dataset

We downloaded proteome sequences from 6,536 fully sequenced bacterial genomes from the KEGG Gene Expression Omnibus (GENOMES) database (Kanehisa et al., 2017). To facilitate the description of the results, we used the KEGG genome identifier, separated by a dash, before each gene identifier.

### Identification of orthologs of BCL in fully sequenced genomes

We first conducted a literature review to find a description of the genes encoding enzymes involved in reactions and pathways, as well as the genomic organization of genes related to BCL. The genomes of *T. aromatica* (*bclA*), *B. xenovorans LB400* (badA), *Azoarcus* spp. (*bzdA*), and *R. palustris* (*badA*) presented experimental evidence of BCL-encoding genes (Egland et al., 1995; Schühle et al., 2003; Kawaguchi et al., 2006; Bains and Boulanger, 2007; Valderrama et al., 2012; Arnold et al., 2021) (Supplementary Table S1). We used this information to search for orthologous sequences of BCLs in bacterial genomes. To this end, we used an approach developed by our group (Martinez-Amador et al., 2019; Soto-Avila et al., 2021), in which the first step was the identification of *Pfam* domains found in the PfamA database (Mistry et al., 2021), whose matrices were used to inspect the amino acid sequences of proteomes downloaded from the KEGG database. Once the *Pfam* domains were identified, we constructed protein architectures. A protein architecture consists of one or more nonoverlapping domains covering a maximum length of a protein. Homolog searches were then performed using the matrices describing the *Pfam* domains. Hits with an *e* value ≤0.001 were retained. These hits were filtered to retain those with a predefined genomic context. Genes encoding the lower metabolic pathways for converting benzoyl-CoA are part of the genomic context. These pathways are often arranged as operons (Egland et al., 1995; Porter and Young, 2014; Arnold et al., 2021). Therefore, instead of considering three upstream and downstream genes, as proposed in our previous work (Martinez-Amador et al., 2019; Soto-Avila et al., 2021), we extended the inspection of genes in the genomic context to 10 upstream and 10 downstream genes from the BCL. The protein architectures and the *Pfam* accession numbers of the genes represented in the genomic context are shown in Supplementary Table S1. As in our previous work (Martinez-Amador et al., 2019; Soto-Avila et al., 2021), for these proteins, we retained hits with an *e*-value cutoff ≤0.001.

### Searching of ungapped motifs

The program MEME (Bailey et al., 2015) was fed with nonredundant homologs of the BCL sequences to construct conserved ungapped motifs. The nonredundant BCLs were selected by running CD-HIT with the default parameters (Fu et al., 2012). If CD-HIT did not select an experimentally characterized BCL in the corresponding cluster, we changed the suggested sequence to one reported experimentally (Supplementary Table S2). We ran the MEME program, asking for motifs with a minimum length of six amino acids and a maximum length of 30, asking for at least eight matrices. These lengths, as well as the number of motifs requested, accounted for the reported motifs found in other class I, subclass Ib aryl-CoA ligases (Clark et al., 2018; Arnold et al., 2021; Muroski et al., 2022). The resulting matrices were analyzed by the MAST program (Bailey and Gribskov, 1998), which scans BCL homologs with conserved protein architecture (AMP-binding AMP-binding_C) and the suggested genomic context.

### Percentage of GC per gene

We downloaded the gene nucleotide sequences of 6,536 genomes from June 2022 from the KEGG database purchased by our group. The GC percentage was calculated as follows: Count (G + C)/Count (A + T + G + C) × 100%.

### Phylogenetic reconstruction

Sequences were aligned with MUSCLE 5 (Edgar, 2022) and trimmed utilizing the default parameters (Capella-Gutiérrez et al., 2009). To construct the tree, we utilized IQ-TREE multicore version 1.6.12 for 64-bit Linux (maximum likelihood) regression with aLRT correction (a standard likelihood ratio test approximation) and 1,000 replicates to identify the optimal bootstrap distance model (Nguyen et al., 2015). Six sequences were selected as a tree outgroup. The sequences were taken from aryl-CoA ligases used in Arnold et al. (2021) original work, which used these sequences to construct a neighbor-joining phylogenetic tree of amino acid sequences from the adenylate-forming enzyme superfamily. Our ultimate tree, which grouped 148 sequences, was visualized using the iTol tool (Letunic and Bork, 2021).

## Results

### Prediction of orthologs of BCL

**Figure 1:**
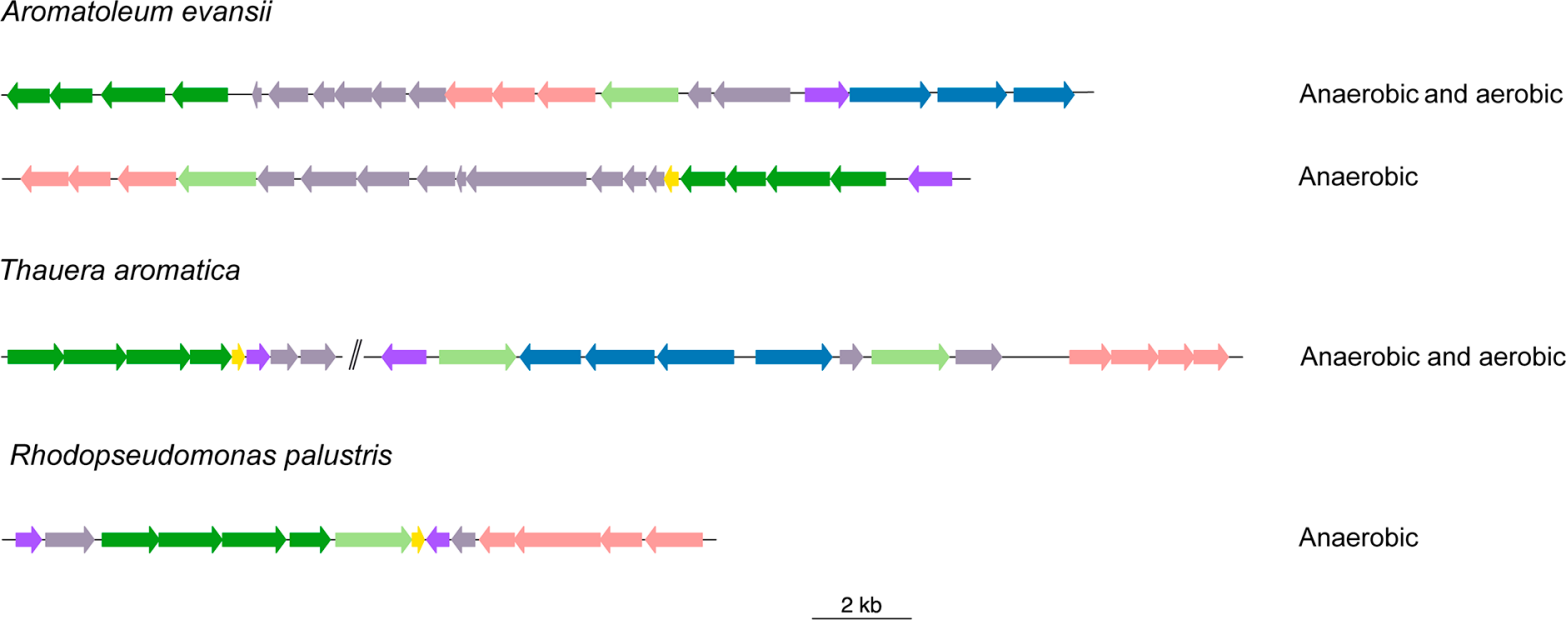
Organization of gene clusters involved in the anaerobic or/and aerobic catabolism of BCLs. Genomic organization of BCL clusters found in *Azoarcus evanssii*. *Rhodopseudomonas palustris* and *Thauera aromática*. The genes are represented by arrows: light green, genes encoding benzoate-CoA ligases; dark green, genes encoding the subunits of the corresponding reductases for anaerobic degradation; yellow, genes encoding ferredoxin; violet, genes encoding the transcription factor; pale rose, genes of ABC transport genes; blue, genes encoding a de *box* cluster; and gray, genes encoding other known or unknown functions. The two vertical lines indicate that the genes are not adjacent in the genome. The figure was constructed using the genoPlotR software with the “gbk” files downloaded from NCBI displaying the genomic contexts.

Identification of BCL orthologs involves multiple steps. One of these steps utilizes the genomic context surrounding BCL homologs to select proteins encoded by genes that maintain a similar context to that observed in the genomes of organisms possessing an experimentally characterized functional BCL. The genomic context of the reported bacteria revealed three types of genomic organization (Figure 1). The genome of *Azoarcus evassii* KB740, now known as *Aromatoleum evansii* KB740 (*A. evassii* KB740), shows two clusters at different positions in the genome (Figure 1). The first cluster was associated with aerobic degradation. The genes included the *boxA* (KO: K15511) and *boxB* (KO: K15512) genes, which encode benzoyl-CoA-2,3-epoxidase enzymes; a *boxC* gene, which encodes benzoyl-CoA-dihydrodiol lyase (KO: K15513); and *boxR*, a gene encoding a transcriptional regulator of the XRE family identified in the KEGG orthologs database by KO: K15546. Additionally, a cluster of benzoyl-CoA reductase subunits (EC:1.3.7.8, KOs: K04112, K04113, K04114, K04115), was also observed. Literature has also shown the presence of three genes encoding ABC transporters. However, the 3,4-dehydroadipyl-CoA semialdehyde dehydrogenase encoded within *boxD* (KO: K15514) was not detected. The second cluster included benzoyl-CoA reductase subunits (EC:1.3.7.8, KOs: K04112, K04113, K04114, K04115) involved in anaerobic degradation. The genes encoding these reductases are regulated by the product of *bzdR* (BzdR), which has the same protein architecture as BoxR and the same KEGG orthology group (KO: K15546). Other bacteria, such as *T. aromatica*, possess two copies of BCL, which are encompassed by cluster housing *box* genes and a group of putative ABC transporters. These proteins are identified as permeases of the branched-chain amino acid transport system. Moreover, the genomes of these bacterial species contain an entire cluster that encodes the subunits of benzoyl-CoA reductase (EC:1.3.7.8), located 179,207 base pairs away from the second copy (tak-Tharo_1138). This demonstrated the ability of *T. aromatica* to metabolize benzoate and related MACs both aerobically and anaerobically, which has also been demonstrated experimentally (Schühle et al., 2003). The BCL of the Alphaproteobacteria *R. palustris* has also been experimentally described. The BCL family has neighboring benzoyl-CoA reductase subunits, a group of putative ABC transporters (Egland et al., 1997) and the transcriptional regulator BadM (Peres and Harwood, 2006). These bacteria possess a cluster of ABC transporters near the BCL, which are not considered components of the lower pathway.

After identifying the protein architecture of the BCLs and the proteins within the genomic context of experimentally characterized genomes, our next step was to recognize homologs of the BCL by utilizing its protein architecture composed of the domains AMP_binding and AMP_binding_C. We found 234 homologs based on this protein architecture (Supplementary Table S3). Among these, 148 homologs retained at least one of the proteins found in the genomic context. Upon closer examination of these 148 homologs, it became apparent that 13 were missing at least one of the genes encoding a subunit of benzoyl-CoA 2,3-epoxidase A or B. This deficiency imposed a new criterion for selecting BCL orthologs. We decided to consider homologs with an incomplete context these 13 proteins, which lack one of the benzoyl-CoA 2,3-epoxidase subunits. Notably, the 13 homologs maintained an ortholog of the transcriptional regulator BoxR within their context (Supplementary Table S4). Adhering to these additional criteria, we ultimately identified 135 homologous proteins with a conserved context. The conservation of a group of ungapped motifs was imposed as a final constraint for predicting BCL orthologs, as calculated using MEME (Bailey et al., 2015). BCLs share six motifs with other class I aryl-CoA ligases in subclass Ib. The remaining two motifs in the N-terminus are characteristic of BCLs (Table 1). The final rules for predicting BCL orthologs are: (a) preservation of the proposed Pfam protein architecture, (b) a valid genomic context, and (c) the conservation of the eight ungapped MEME motifs in the order found in the experimentally characterized BCLs. Sequences adhering to these criteria were categorized as FC_FMs (complete context and full motifs), considering that all examined sequences possess the proposed BCL protein architecture. The detailed predictions of the motifs are shown in Supplementary Table S5. These rules yielded 132 BCL sequences distributed in 118 bacteria, showing that our method still leaves indistinguishable paralogs.

**Table 1:** Conserved motifs of the predicted BCL orthologs.

From N to C-terminal	MEME motif	Described motif function	Reported motifs (aryl-CoA-ligases)	Reference
BCL-motif-7	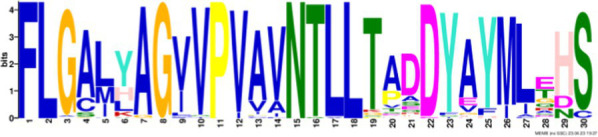	Unknown function	—	This work
BCL-motif-1	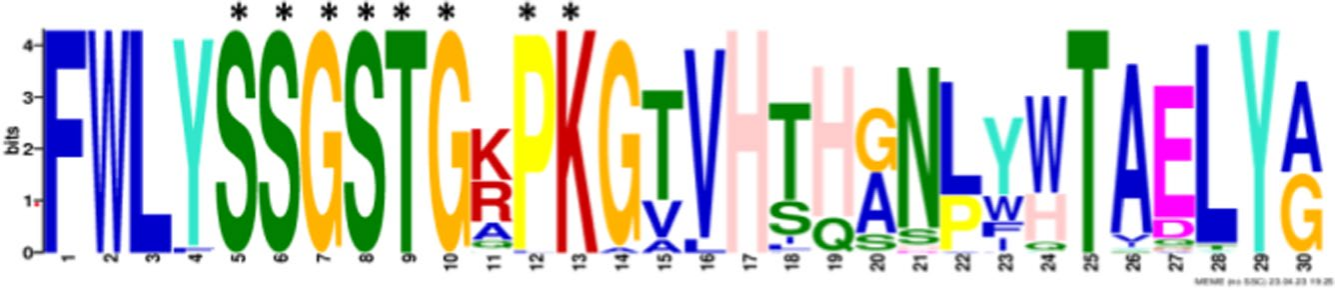	P-loop; regulates the interaction and binding of phosphate with GDP	YYx**(S/T)(S/T/G)G(S/T)**TGxPK	Arnold et al. (2021)
BCL-motif-6	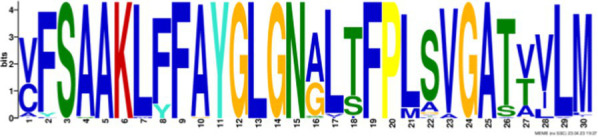	Substrate recognition	Ala227	This work
BCL-motif-8	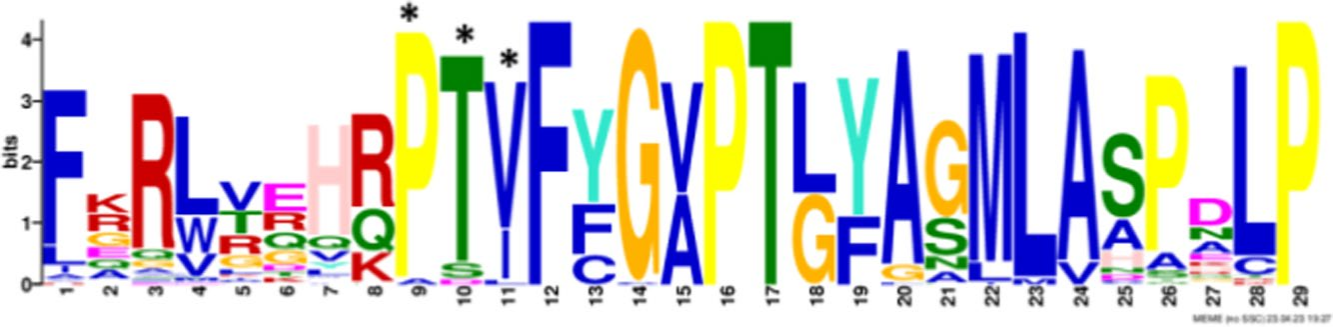	Pro278; ATP positioning and Mg^2+^ binding	(I/L)(E/Q)K(Y/E)(K/R)(V/I)Tx(L/F)xG(V/A)**PTIYR**(F/A)L(L/A)(K/Q)	Clark et al. (2018)
BCL-motif-2	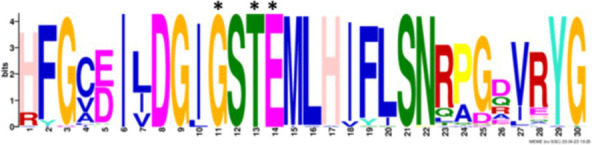	ATP positioning and Mg^2+^ binding	W**(G/W)**x**(A/T)E**	Arnold et al. (2021)
BCL-motif-4	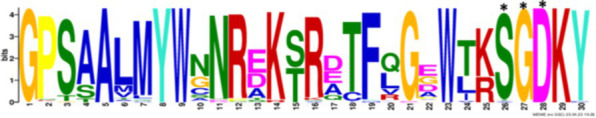	Unknown function	**(S/T)GD**	Clark et al. (2018) and Arnold et al. (2021)
BCL-motif-3	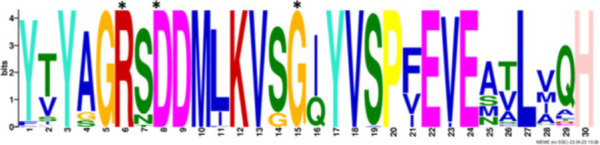	ATP positioning, binding/interaction	**Rx(D**/K)x6*G* (Lys427) from *R. palustris*	Thornburg et al. (2015)
BCL-motif-5	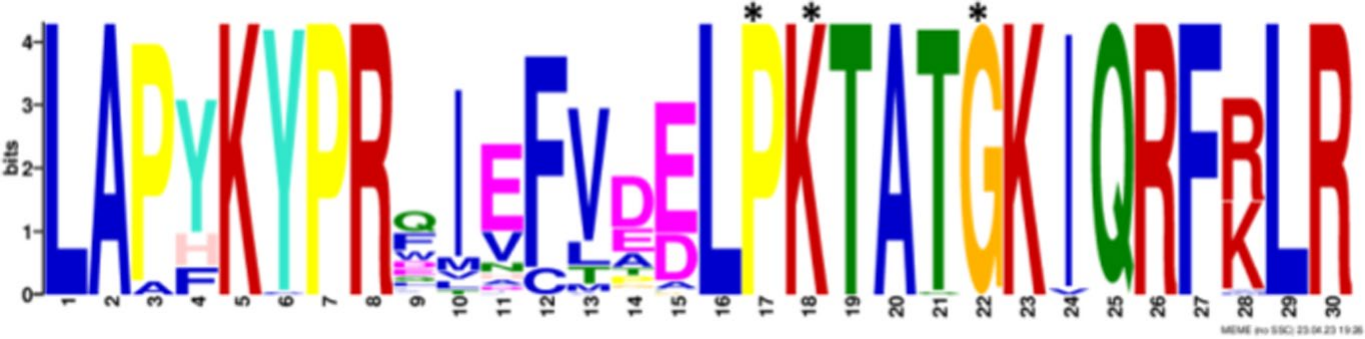	It interacts in the adenylation conformation	Ω**(G/W)x(A/T)E**	Arnold et al. (2021)

The orthologous sequences of the BCLs retained eight ungapped motifs. Five motifs must interact with the benzoate molecule and the active site. An asterisk in the logo indicates the residues that match motifs identified in other works, highlighted in bold. “Ψ,” aromatic amino acid (F, Y, H, or W); “Ω,” aliphatic amino acid (A, V, L, I, or M); x, any amino acid.

The second group of homologs included proteins with an invalid context (IC_FM, incomplete context but full motifs). For some of these proteins, a copy of the BCL was predicted and grouped into FC_FM. We considered the copies found in both groups to be paralogs. In the third group, we found copies or unique proteins that presented a complete context and a duplication of one of the ungapped motifs (FC_DM). The protein encoded by the eba-ebA2757 gene has one copy (eba-ebA5301) in the genome of *Aromatoleum aromaticum* EbN1 (*A. aromaticum* EbN1), grouped into FC_FM. In a recent study, the presence of paralogs of BCL in *A. aromaticum* EbN1 was reported (Suvorova and Gelfand, 2019). In this work, the authors showed that one copy colocalizes with *box* genes. The other colocalized with the anaerobic benzoate metabolism gene cluster, a result reproduced in our study.

### Structural features and conserved residues of benzoate-CoA ligases to improve the search for BCL orthologs

We constructed MEME motifs to improve the ortholog search. Other works have described MEME motifs found in the adenylate-forming enzyme superfamily Class I (Marahiel et al., 1997; Clark et al., 2018; Arnold et al., 2021), which includes groups of conserved residues and structural features consistent with our results. A comparison of our predicted motifs with the reported motifs showed that BCL-motif-1 is located in the N-terminus and is associated with a phosphate loop (P-loop). The motif found in aryl-CoA ligases has the following consensus sequence: YYx(S/T)(S/T/G)G(S/T)TGxPK (Arnold et al., 2021). We found this consensus in BCL-motif-1, with 100% conservation of Ser{181}, Ser{182}, Gly{183}, Ser{184}, Thr{185}, Gly{186}, Pro{188} and Lys{189}, using the RPA066 gene of *Rhodopseudomonas palustris* BisB5 (*R. palustris* BisB5) as a reference (Table 1). BCL-motif-2 is similar to the sequence reported in other aryl-CoA ligases and is important for ATP and Mg^2+^ binding (Table 1). From the reported consensus [W(G/W)x(A/T)E], we observed that the residues Gly{327}, Ser,{328} Thr{329} and Glu{330} are fully conserved in the BCL sequences (marked with * in Table 1). The ANL superfamily contains a motif similar to that of BCL-motif-8, in which the PTIYR residues are fully conserved (Table 1); however, BCL-motif-8 has a fully conserved Pro{269}, but instead of a 100% conserved (Arg), Phe{272} is preserved in the motif in 100% of the sequences. BCL-motif-3 resembles the Rx(D/K)x6G motif (Thornburg et al., 2015), in which we observed that Arg{421}, Asp{423}, and Gly{430} are fully conserved. BCL-motif-3 is located in the C-terminal domain and contacts the outer edge of the benzoate binding pocket, positioning benzoate in the active site through a charged interaction between the carboxylate group of benzoate and Lys{427} of *R. palustris* BisB5. According to the consensus sequence found for BCL-motif-3, the Asp{424}, Met{425}, and Val{428} residues near the Lys{427} contact site are fully conserved. BCL-motif-5 is located at the C-terminus and retains Lys{512}, which is the key catalytic residue that interacts in the adenylation conformation of the enzyme with alpha-phosphate during nucleophilic attack on ATP. Lys{512} is highly conserved in the aryl-CoA ligases acyl-CoA and fatty acid acyl-CoA (Arnold et al., 2021). BCL-motif-4 shares a conserved Asp{406} with aryl-CoA ligase and Gly{405}, which has been reported to be conserved in BCLs, the 4-hydroxybenzoate-CoA ligase of *T. aromatica* and *R. palustris*, the 3-hydroxybenzoate-CoA ligase of *T. aromatica* and the 2-aminobenzoate-CoA ligase of *A. evansii* (Arnold et al., 2021). The motifs BCL-7 and BCL-6 identified in this study are located in the N-terminus, and both of these motifs have unknown functions. BCL-motif-6 contains a strongly conserved region (FAYGLGN), and BCL-motif-7 contains a conserved Asp region (Table 1).

### The genomic context is a powerful tool for predicting the oxygen requirements for benzoyl-CoA degradation

**Figure 2:**
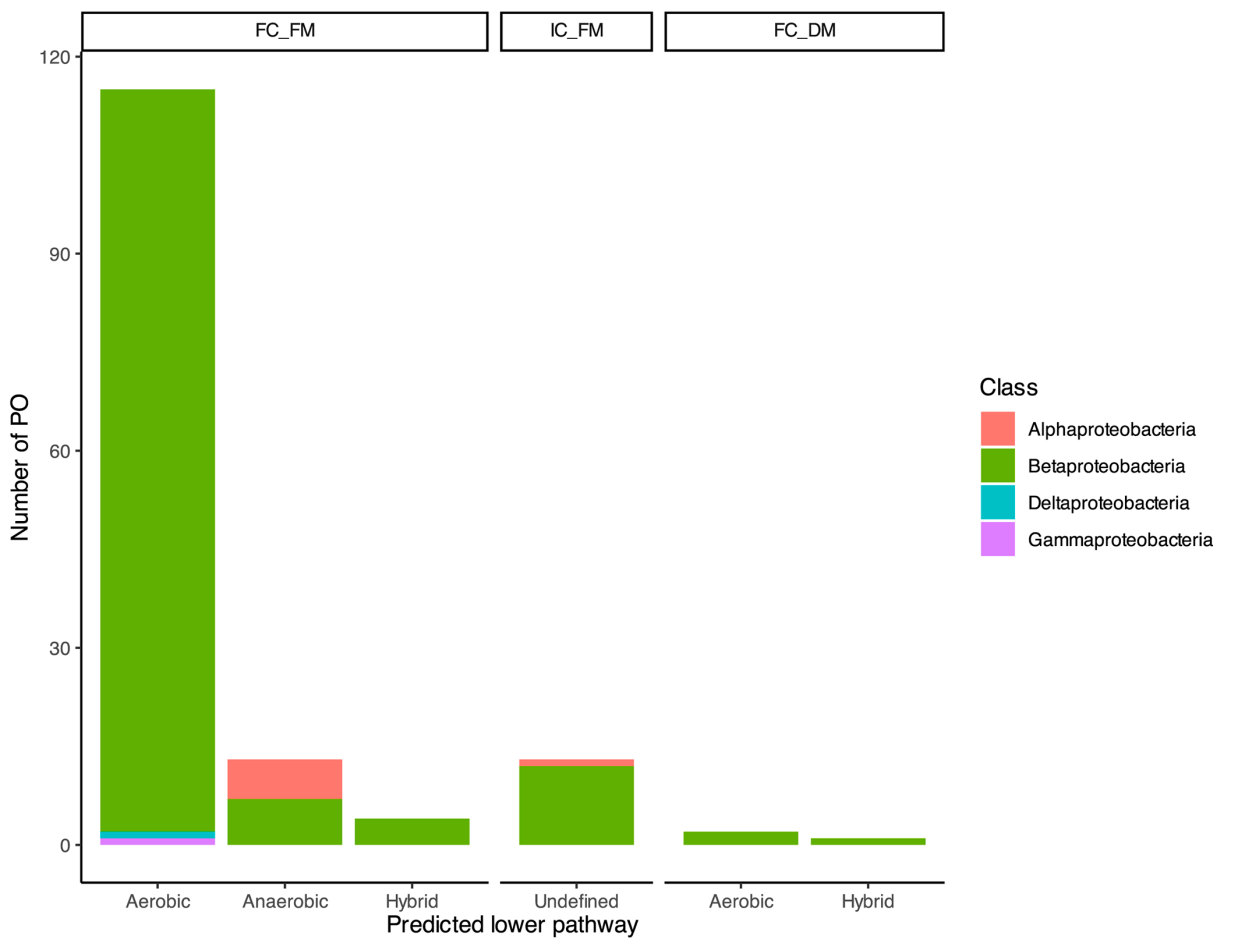
Taxonomic distribution of the predicted BCL orthologs. Based on their agreement with the proposed prediction steps, the bar plot illustrates the distribution of 148 probable orthologs of BCL across taxonomic classes. The bars provide insights into the potential benzoyl CoA lower degradation pathway, distinguishing between aerobic, anaerobic, or hybrid pathways.

The genomic context plays a crucial role in identifying not only probable orthologs (POs) of BCL but also the presence of reductases and epoxidases in the vicinity, which could indicate whether subsequent reactions catalyze the conversion of benzoyl-CoA through an aerobic or anaerobic pathway. Within the 132 sequences classified as FC_FM, nine POs had enzymes involved in anaerobic metabolism in their genomic context; 113 POs exclusively had enzymes involved in aerobic degradation, and 10 POs could proceed through aerobic and anaerobic pathways, which will refer to catabolism as hybrid catabolism. Among these 132 sequences, Betaproteobacteria retained 94% (sequences = 124) of the hits, which were distributed as follows: 113 associated with aerobic pathways, seven with anaerobic pathways, and four involved in hybrid catabolism. In the case of Alphaproteobacteria, the genomic context suggested that the six POs may be involved in an aerobic degradation pathway. Additionally, we predicted one PO of BCL in Deltaproteobacteria and another in Gammaproteobacteria; in both cases, the lower pathway suggested aerobic catabolism. The IC_FM comprises 13 sequences, with 12 distributed in Betaproteobacteria and one in Alphaproteobacteria. These sequences produced an undefined phenotype, as we could not predict the lower pathway with the available context information (Figure 2). Within the FC_DM, three Betaproteobacteria sequences were enclosed. Two exhibited a hybrid genomic context, while the remaining showed an aerobic context (Figure 2).

**Figure 3:**
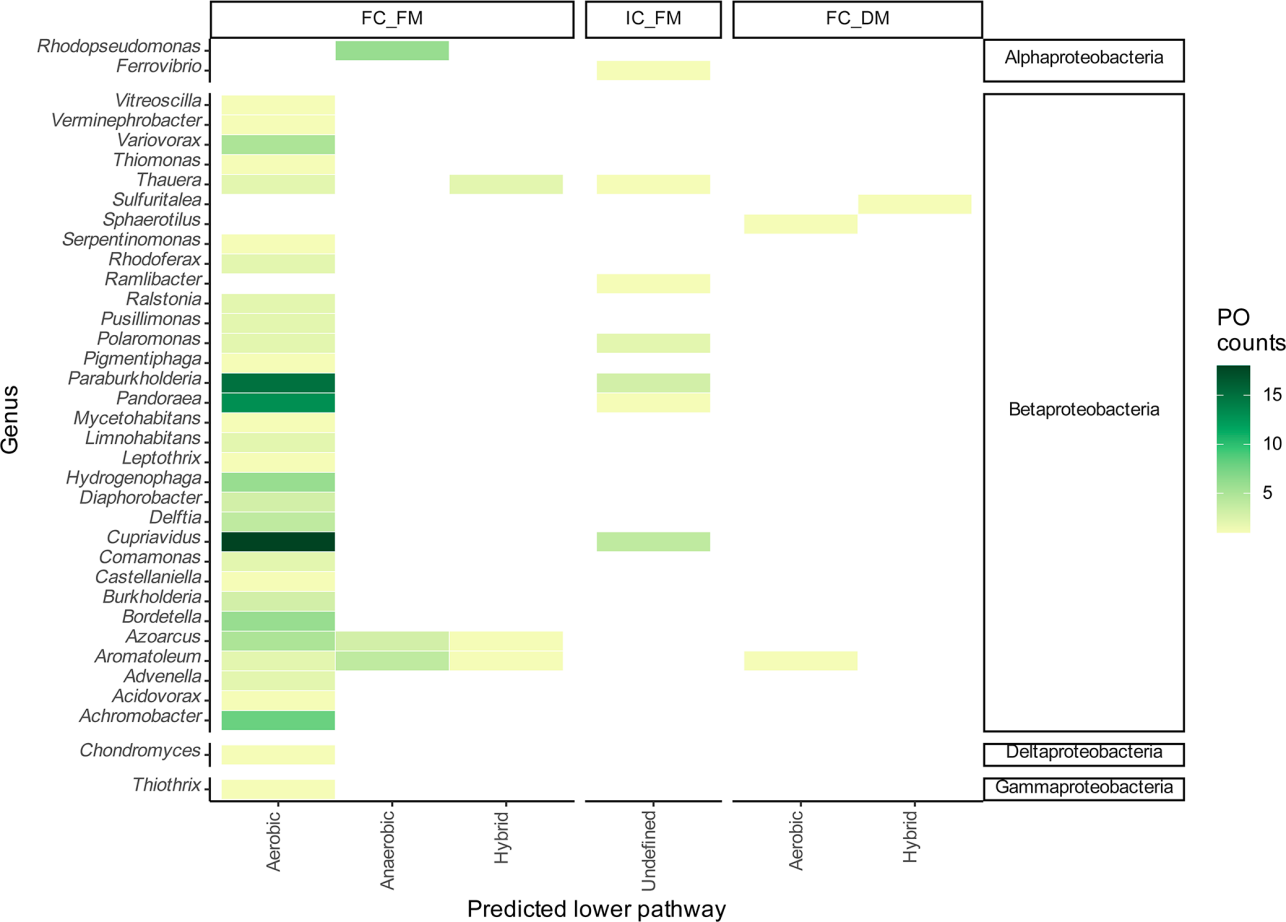
Taxonomic distribution of probable orthologs of BCLs within the genera. The heatmaps represent the frequency of observed probable orthologs of the BCL by genus as a function of the followed prediction steps.

The FC_FM group comprises 32 taxonomical genera with 29 protein sequences distributed among Betaproteobacteria. *Bordetella* was represented by six sequences, constituting 4.55% of the total. *Hydrogenophaga* contributed six sequences (4.55%), *Achromobacter* included eight sequences (6.06%), *Pandoraea* included 13 sequences (19.85%), *Paraburkholderia* included 16 sequences (11.35%), and *Cupriavidus* included 20 sequences (13.64%). These genera reveal the potential for an aerobic lower pathway, which was the more prevalent lower pathway found in the inspected proteomes (Figure 3). The genus *Thauera* contains four POs of the BCL associated with two potential aerobic pathways and the same number of potential hybrid lower pathways. *Ralstonia* contains two POs, the genomic context of which indicates potential aerobic degradation of benzoyl-CoA. *Azoarcus*, with nine POs representing 6.82% of the total sequences analyzed, exhibited diverse genomic contexts: five proteins with a potential aerobic context, three with an anaerobic context, and one with a hybrid genomic organization. Notably, three *Azoarcus* genomes exhibited two predicted copies of the BCL. We also observed copies of the BCL in the genomes of 15 bacteria distributed among the genera *Cupriavidus*, *Thauera*, *Ralstonia*, *Paraburkholderia* and *Aromatoleum*, which were classified as indistinguishable paralogs since all the POs possess one of the proposed genomic contexts.

IC_FM grouped sequences with incomplete Box clusters in their genomic contexts, classifying them as sequences with undefined lower pathways. IC_FM consisted of one protein from *Ferrovibrio terrae* K5, an Alphaproteobacteria member, and 12 Betaproteobacteria distributed among the genera *Cupriavidus*, *Pandoraea*, *Paraburkholderia*, *Polaromonas*, *Ramlibacter*, and *Thauera* (Figure 3).

Three sequences showed a fully conserved context but exhibited redundancy with one of the MEME motifs, a group labeled FC_DM (Figure 3). These sequences are present in the genomes of *A. aromaticum* EbN1, which contains two copies of BCLs, both of which are functional (Rabus et al., 2014). One of the copies, eba-ebA2757, has an extra BCL-motif-8 in the N-terminus with an ATP position and a Mg^2+^ that is poorly conserved except for the catalytic proline Pro{269}. The shd-SUTH_01659 protein from *Sulfuritalea hydrogenivorans* has all the expected motifs, with duplication of BCL-motif-2 in the N-terminus. On the other hand, snn-EWH46_00425 from *Sphaerotilus natans* subsp. *sulfidivorans* D-507 had an extra copy of BCL-motif-3 in the N-terminus (Supplementary Table S5). The detailed phyletic profile and taxonomy of the predicted POs of the BCLs grouped in the FC_FM, IC_FM and FC_DM groups can be found in Supplementary Table S4.

### Taxonomic distributions of paralogous sequences

**Figure 4:**
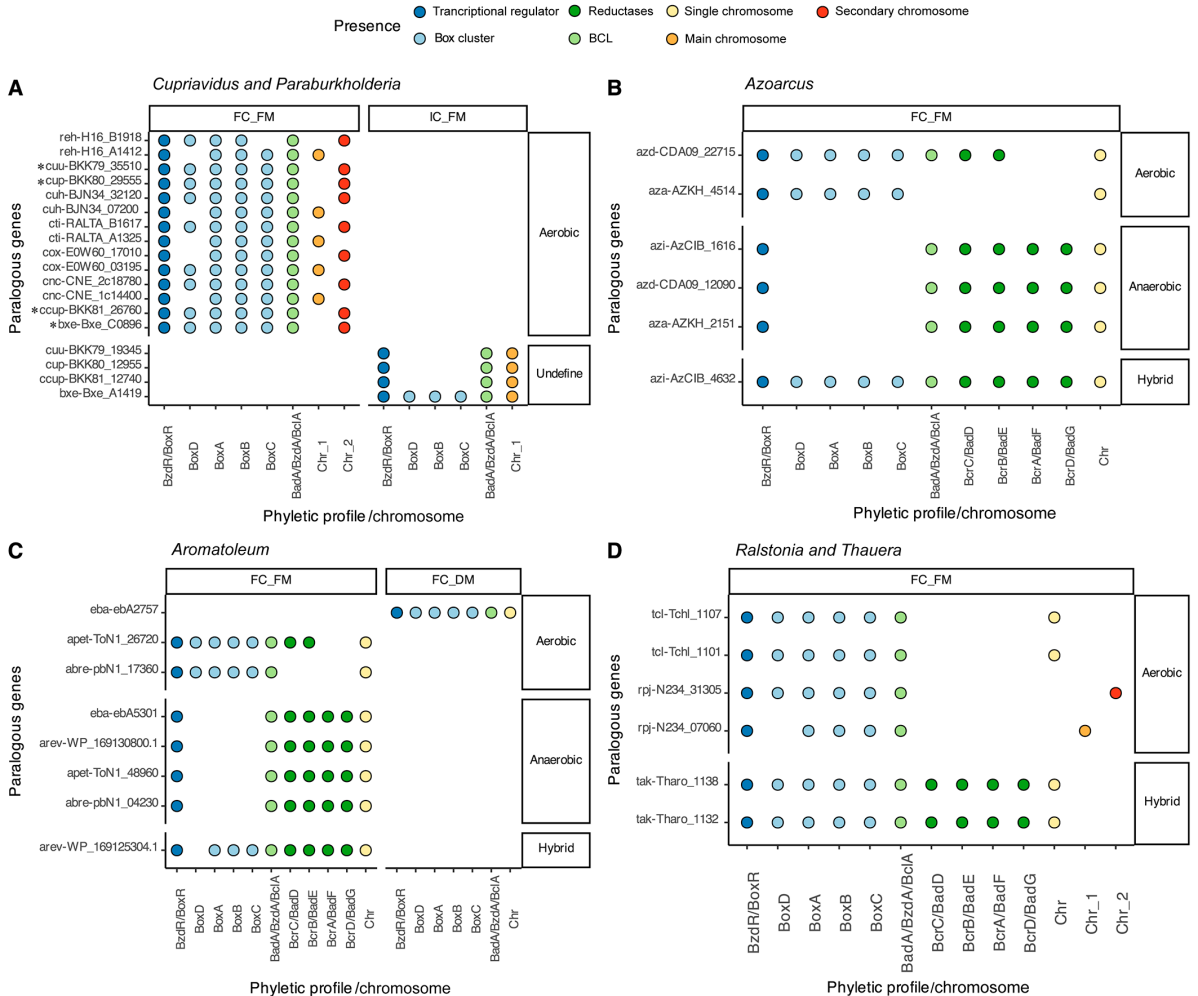
Distribution of paralogous copies of the BCL within the genus. Each panel displays the phyletic profile of indistinguishable paralogs of benzoate CoA ligases, along with their positions within and outside multipartite genomes. **(A)** *Cupriavidus* and *Paraburkholderia*; **(B)** *Azoarcus*; **(C)** *Aromatoleum*; and **(D)** *Ralstonia* and *Thauera*. Genes marked by an asterisk are probable orthologs according to the proposed recognition steps.

In some genomes, differentiating between paralogous copies and orthologs poses a challenge, as all the predicted BCLs adhere to the proposed prediction steps. This was evident in the case of five *Cupriavidus* genomes, where the predicted BCLs retained a Box cluster in their context, as did the eight motifs characterizing a BCL in this work (Figure 4A). On the other hand, three other *Cupriavidus* genomes, as shown in Figure 4A, featured one BCL copy not adhering to all the detection criteria (IC_FM), since they exhibited an incomplete context, retaining only a PO of the transcriptional regulator BoxR.

As mentioned, all BCLs with an incomplete context and a complete set of motifs were categorized in the IC_FM group, suggesting that these copies are not orthologs of the BCL. Upon scrutiny of these genomes, it became apparent that copies in the IC_FM group are encoded in the main chromosome, while those in the FC_FM group are encoded in a secondary chromosome (Figure 4A), making ortholog identification difficult. The same pattern was observed in the genome of *P. xenovorans* LB400, in which the copy preserving a full aerobic context was encoded in a megaplasmid, whereas the copy lacking a full context was encoded in the genome (Figure 4A).

Three genomes of *Azoarcus* encoded indistinguishable paralogous copies, all of which were classified in the FC_FC group. Specifically, the azd-CDA09_12090 and azi-AzCIB_1616 genes of the species *Azoarcus* sp. DN11 (*A.* sp. DN11) and *Azoarcus* sp. CIB (*A.* sp. CIB), respectively, feature a gene cluster involved in anaerobic degradation in their genomic contexts (Figure 4B). The second set of genes, azd-CDA09_22715 (*A.* sp. DN11) and azi-AzCIB_4632 (*A.* sp. CIB), contained a cluster of Box proteins and orthologs of the transcriptional regulator BoxR in their genomic contexts. The azd-CDA09_22715 protein also exhibited orthologs encoding the proteins BcrA (K04114) and BcrD (K04115) from the anaerobic cluster (Figure 4B). Despite these bacteria containing BcrA and BcrD, we classified them as aerobic due to the prediction of a complete aerobic pathway for benzoyl-CoA degradation and the absence of the remaining reductase subunits, suggesting that they cannot activate the anaerobic pathway.

Seven indistinguishable paralogous copies identified in *Aromatoleum* were categorized in the FC_FM group, while one was placed in the FC_DM group. Nevertheless, as illustrated in Figure 4C, each paralog exhibited a distinct genomic context from its counterpart. A similar pattern is evident in the sequences of the genus *Azoarcus*, as depicted in Figure 4B, where sequences with potential aerobic, anaerobic, or hybrid degradation pathways are observed.

*Thauera* exhibited two species with indistinguishable paralogous copies, all of which were classified in the FC_FM group. Two of these copies potentially proceed through an aerobic benzoyl CoA degradation pathway, while the other two may catabolize the substrate via an anaerobic pathway, as depicted in Figure 4D. The *Ralstonia pickettii* DTP0602 paralog includes two POs of the BCL (rpj-N234_07060 and rpj-N234_31305) with a potentially aerobic pathway.

### Phylogenetic distribution of orthologs and paralogs

The inability to distinguish orthologs from paralogs in some species motivated us to construct a rooted phylogenetic tree that should elucidate the origins of these copies. For this purpose, we used a set of six class I subclass Ib aryl-CoA ligases from the study of Arnold et al. (2021), as described in the Methods section, as well as the sequences of the predicted orthologs and paralogs of the BCLs, which were categorized into three groups: FC_FM, IC_FM, and FC_DM.

#### Phylogenetic distribution of paralogs

**Figure 5:**
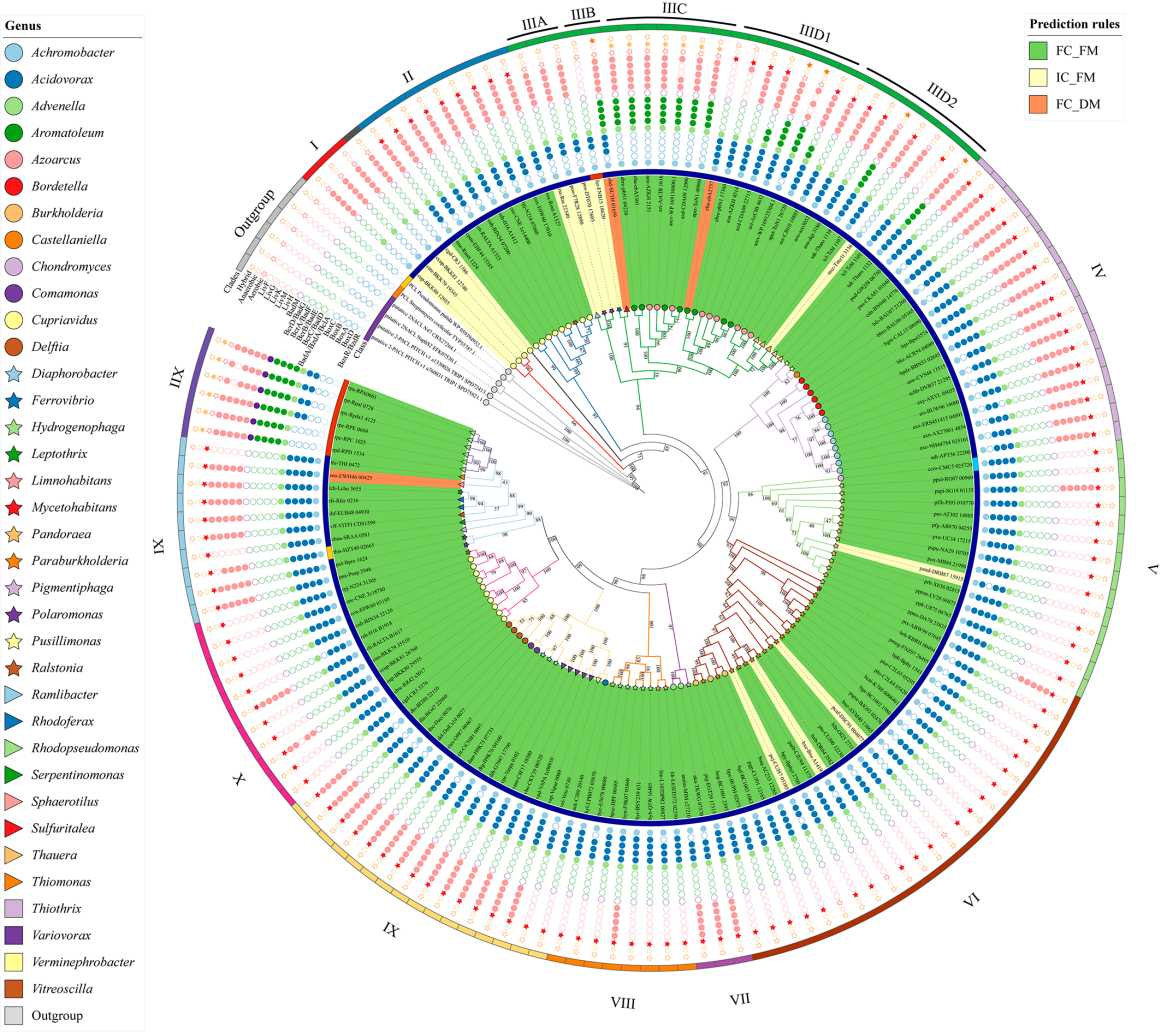
Phylogenetic distribution of orthologs and paralogs of BCL. The tree has been segmented into twelve clades, each distinguished by Roman numerals. Phyletic profiles display empty dots indicating absences and filled dots indicating presence. Stars denote phenotypes. Geometric figures at each tip represent BCLs within genera. Taxonomical genera, classes, and BCLs clustered by FC_FM, IC_FM, and FC_DM are also presented.

**Figure 6:**
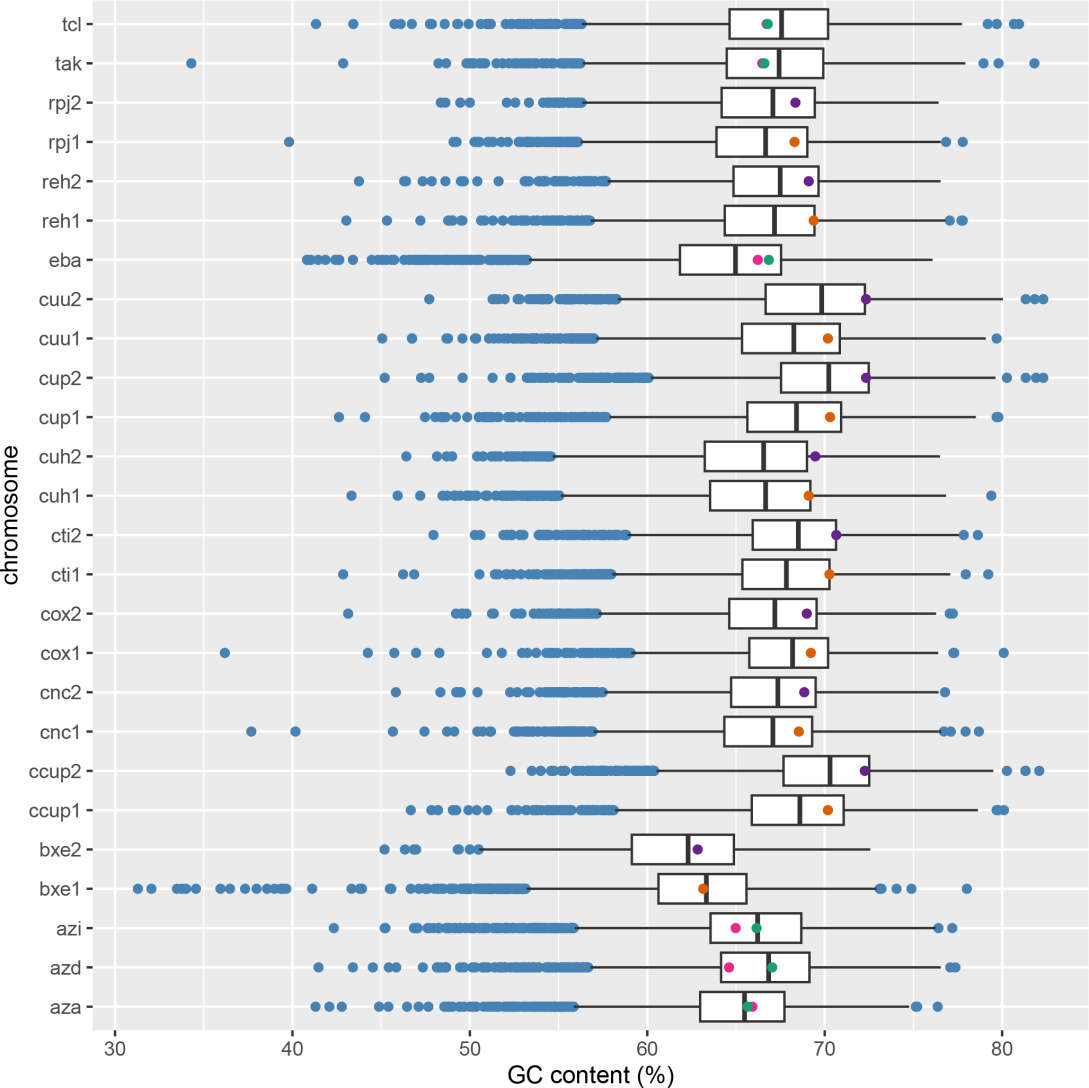
The distribution of %GC in genomes featuring paralogous copies of the benzoate-CoA ligase (BCL). In multipartite genomes, orange dots indicate BCLs on the main chromosome, while purple dots represent BCLs on megaplasmids or chromids. The green and pink dots for BCLs indicate copies encoded on the same chromosome. The *y*-axis labels include tcl for *Thauera chlorobenzoica* 3CB1, tak for *Thauera aromatica* K172, rpj for *Ralstonia pickettii* DTP0602, reh for *Cupriavidus necator* H16, eba for *Aromatoleum aromaticum* EbN1, cuu for *Cupriavidus* sp. USMAA2-4, cup for *Cupriavidus malaysiensis* USMAA1020, cuh for *Cupriavidus necator* NH9, cti for *Cupriavidus taiwanensis* LMG 19424, cox for *Cupriavidus oxalaticus* X32, cnc for *Cupriavidus necator* N-1, ccup for *Cupriavidus* sp. USMAHM13, azi for *Azoarcus* sp. CIB, azd for *Azoarcus* sp. DN11, and aza for *Azoarcus* sp. KH32C, bex for *Paraburkholderia xenovorans*. LB400, with 1 referring to the main chromosome and 2 to the secondary chromosome (chromid or megaplasmid).

BCL paralogs were identified in the *Aromatoleum*, *Azoarcus*, *Cupriavidus*, *Paraburkholderia*, *Ralstonia*, and *Thauera* genera. The phylogenetic tree in Figure 5 highlights a specific group of copies originating from *Cupriavidus* and *Ralstonia*, near the root. These paralogs formed two distinctive clades, clades I and II. Clade I comprises four paralogs organized into the IC_FM group, whereas clade II includes eight *Cupriavidus* paralogs and one *Ralstonia* paralog, grouped as FC_FM. The paralogs in clade II share an aerobic phyletic profile and exhibit a cluster of POs associated with ABC transporter proteins. The second set of BCL copies, similar to those found in clades I and II, were collectively clustered into clade X, characterized by an aerobic phyletic profile. The genes encoding cti-RALTA_B1617 and cup-BKK80_29555 grouped into clade X displayed phyletic profiles with POs related to ABC transporter proteins, a pattern frequently observed in experimentally studied genes. The genes organized into clades I and II are encoded on the main chromosome, and those in clade X are encoded on a second chromosome (chromid). To determine whether the nucleotide composition of the BCLs in a second clade was like that of their genomes, we conducted a comparison between the %GC of the BCL and that of its related chromid or chromosome (Figure 6). Those in clade I are encoded on the chromosome, with %GC within the interquartile range. Its counterparts encoded by chromids grouped in clade X have BCLs in the upper quartile (q3) border, indicating a recent acquisition. Notably, the median length of the second chromosome exceeded that of the main chromosome, indicating the recent arrival of several genes on the second chromosome. These genera share a bordering copy in q3, except for cuh2 (cuh-BJN34 32120), which is an outlier (Figure 6). Notably, the BCLs on the primary and secondary chromosomes fell within the interquartile range of q3 or near its upper limit in these bacteria with high %GC, with a median of 68%–70% (Figure 6).

Clades IIIC and IIID1 contained bacteria of the genera *Aromatoleum* and *Azoarcus*. The POs of the BCLs grouped in clade IIIC showed a phyletic profile consisting of a complete set of reductases and a cluster of ABC transporters, suggesting the conversion of benzoyl-CoA by anaerobic catabolism. However, clade IIID1 had two copies (eba-ebA2757 and abre-pbN1_17360) capable of metabolizing benzoyl-CoA through the aerobic Box pathway. Notably, the product of eba-ebA2757 is a BCL with a duplicated motif in the N-terminus. The phyletic profile of the remaining copies of clade IIID1 suggested hybrid metabolism in which the Box proteins and the subunits of the benzoyl-CoA reductase were present. On the other hand, the phyletic profiles of azd-CDA09_22715 and apet-ToN1_26720 lack the D and A subunits of benzoyl-CoA encoded by *bcrD*/*badG* and *bcrA*/*badF*, suggesting an aerobic catabolism. The copies found in *Thauera* were divided into two subclades: one involved in hybrid catabolism and the other involved in aerobic catabolism (Figure 5). The copies within the members of *Azoarcus* are encoded on the same chromosome (Figure 4), and as shown in Figure 6, they are grouped inside the interquartile range. These species with a %GC distributed near the median belonged to clade IIID1, which was located farther from the root. Two copies within clade IIIC (Figure 5) were distributed within quartile 1 (azi-AzCIB_1616 and azd-CDA09_12090), and a third one was distributed within quartile 3 (aza-AZKH_2151) (Figure 6), suggesting that BCLs associated with hybrid and aerobic degradation found in bacteria within clade IIID1 are better adapted to the genome. Notably, clade IIIC and IIID1 had a recent common ancestor, indicating that both BCLs were acquired during recent events. The two BCL copies in *Thauera* were within the interquartile range, exhibiting nearly identical %GC values near the median (Figure 6). This implies that if the second copy was horizontally transferred, it was either acquired near the arrival of the first copy or adapted rapidly to the genome.

Among the Paraburkholderiales examined, *P. xenovorans* LB400 exhibited two BCLs. One copy was encoded into the primary chromosome, and the other was in a secondary chromosome; categorized as megaplasmids. Like in *Cupriavidus*, the copy within the primary chromosome exhibited an incomplete Box cluster, housing both BoxA and the transcription regulator BoxR. Conversely, the copy located in the megaplasmid encodes an entire aerobic Box cluster along with the PO of the transcription regulator BoxR.

#### Phylogenetic distribution of proteins encoded in a single gene copy

Among the 132 predicted proteins clustered in FC_FM, 101 were encoded in a single copy in the respective genomes. We considered these single-copy proteins orthologous to BCL, as they adhere to the proposed prediction criteria. Clade II is a heterogeneous cluster since it clusters single-copy genes and a set of indistinguishable paralogs of the BCL with aerobic genomic contexts. Clade IIIA, by its part, holds four predictions catalog as IC_FM, distributed in the genera *Polaromonas* and *Ferroviario*, where *Ferroviario* has just one representative in the whole set. Clade IIIB has two members in distinct genera, each distributed in the IC_FM and FC_DM groups. As in the case of *Ferrovibrio*, these clades have unique representatives of the genus. Clade IIID2 includes single-copy BCLs of *Azoarcus* and *Thauera*, a feature that distinguishes them from other members of the genus, with two predicted copies of this enzyme encoded in their genomes. *Azoarcus* sequences show an aerobic phyletic profile. In contrast, the BCLs found in *Thauera* exhibited predicted hybrid benzoyl-CoA degradation pathways (Figure 5).

The predicted orthologs within the Alcaligenaceae family formed a cluster within clade IV. These members can potentially catabolize benzoyl CoA through an aerobic lower pathway. The genomic context encompasses POs of the transcriptional regulator (BoxR) and a complete set of POs of the ABC transporters. Additionally, all species within clade IV belong to the FC_FM group, which comprises representatives of the *Achromobacter*, *Bordetella*, *Pusillimonas*, and *Castellaniella* genera.

Some of the predicted BCLs within the Burkholderiaceae family were grouped in clade V, with one found in the genus *Chondromyces* and 12 in the genus *Pandoraea* (Figure 5). The phyletic profile indicates likely aerobic metabolism guided by the presence of POs within the Box cluster. Among these, one protein is categorized as IC_FM, primarily due to the absence of subunit A of the benzoyl-CoA 2,3-epoxidase (BoxA). Interestingly, no observations of POs related to ABC transporters were noted in the genomic context. Additionally, this clade includes the sole predicted BCL in Deltaproteobacteria, featuring an anticipated aerobic Box cluster in its context. Clade VI included representatives from the genera *Paraburkholderia*, *Burkholderia*, *Mycetohabitans*, and *Pandoraea*, with three predicted POs classified in the IC_FM group. Both clades V and VI exhibited a preserved Box cluster, and 99.05% of the species within these clades lacked genomic clusters of ABC transporters (Figure 5).

Two bacteria classified in the genus *Advenella* and one in the *Pigmentiphaga* genus belonging to the family Alcaligenaceae (Supplementary Table S4) were grouped in clade VII. All the bacteria exhibited an aerobic phyletic profile in which the ABC transporter cluster was present. Eight representatives of the family Comamonadaceae were clustered in clade VIII, which included organisms classified in the genera *Hydrogenophaga* and *Limnohabitans*, in which the genomic context suggested aerobic degradation of benzoyl-CoA via the Box pathway (Figure 5). The species *Hydrogenophaga* sp. BPS33, which contributes the protein hyn-F9K07_05860 to the BCL ortholog group, has a complete set of Box proteins and a cluster containing an ABC transporter in the genomic context. In contrast, the BCLs hyc-E5678_00680 and lim-L103DPR2_00827, found in *Hydrogenophaga* sp. PAMC20947 and *Limnohabitans* sp. 103DPR2, respectively, are deficient in a probable ortholog of the 3,4-dehydroadipyl-CoA semialdehyde dehydrogenase encoded by *boxD*. The Comamonadaceae family contains bacteria from the genera Var*iovorax*, *Delftia*, *Diaphorobacter*, *Comamonas*, *Acidovorax*, and *Verminephrobacter*, which are placed in clade IX. As in other clades, the members included in each genus show a phyletic profile related to the aerobic degradation of benzoyl-CoA. The observed profile does not apply to the protein drg-H9K76_04160 found in the genome of *Diaphorobacter ruginosybacter* DSM 27467, which contains a complete set of Box proteins, in contrast to the profile found for Daer-H9K75_07735 of *Diaphorobacter aerolatus* KACC 16536 and dih-G7047_17790 of *Diaphorobacter* sp. HDW4A, both of which lack BoxD (Figure 5). Thus, we observed that the phyletic profile is frequently conserved at the genus level.

Clades XI and XII share a recent common ancestor. However, they split into two subclades at some point in evolution (Figure 5). Clade XI included five Comamonadaceae species; one BCL was predicted to be in the Thiotrichaceae family, and the other was in the Neisseriaceae family (Supplementary Table S4). Two other BCLs belonging to the genera *Leptothrix* and *Thiomonas* were not assigned to any family. This clade contains the only ortholog associated with Gammaproteobacteria (*Candidatus Thiothrix singaporensis* SSD2) found in the genus *Thiothrix.* All the BCLs of clade XI exhibited an aerobic phyletic profile. In this group, we also found *Sphaerotilus natans* subsp. *sulfidivorans* D-507, a third species with a duplicated motif (snn-EWH46 00425). Clade XII features a small group of Alphaproteobacteria with BCL orthologs that displayed an anaerobic benzoyl-CoA degradation pathway. This pathway includes identifying a group of ABC transporters and the presence of the transcriptional regulator BadM, which we only found in the genomic context of Alphaproteobacteria.

## Discussion

This study’s ultimate prediction of orthologs hinges on employing a blend of bioinformatic techniques centered around the conservation of protein domains and the preservation of genomic context. These methods, utilized by our team and other groups, have proven effective in forecasting genomic markers for other biological processes, such as those involved in various stages of the endosporulation process in Firmicutes (Davidson et al., 2018; Kelly and Salgado, 2019; Martinez-Amador et al., 2019; Soto-Avila et al., 2021). Recently, this strategy, complemented by machine learning techniques, was applied to identify proteins of the adenylate-forming (ANL) enzyme family to define major functional classes (Robinson et al., 2020). These classes share the (Y/F)(G/W)X(A/T)E and (S/T)GD motifs critical for ATP binding and catalysis (Marahiel et al., 1997; Clark et al., 2018; Arnold et al., 2021). The ANL classes predicted in the machine learning study were utilized as input to construct a maximum likelihood phylogenetic tree, revealing that the aryl-CoA ligase group was more closely related to distinct protein subfamilies than to those within them. Another study reconstructed a neighbor-joining phylogenetic tree, which aligned 374 protein sequences of the ANL superfamily, 49 of which were aryl-CoA ligases; these proteins conserved five amino acids in all the groups: Glu328, Gly384, Asp418, Arg433, and Lys524 (Clark et al., 2018; Arnold et al., 2021). These residues were found mainly surrounding the active site of the enzymes containing the AMP- and CoA-binding sites rather than the protein surface. Phylogenetic reconstruction of the 374 sequences revealed nine clades that exhibited group-specific conservation and conservation of 10 motifs, nine of which can be found in the broader ANL superfamily (Marahiel et al., 1997; Gulick, 2009). Like the study presented by Robinson et al. (2020), this work encountered challenges in accurately classifying certain ANLs, highlighting the nontrivial nature of ortholog prediction in the ANL family. However, these enzymes exhibit variations in specific motifs and residues, underscoring the significance of these features for more accurate computational identification of orthologs. In this study, we demonstrated that this refinement enhanced the recognition of BCLs from other aryl-CoA ligases.

Our analysis of the motifs revealed eight conserved motifs, two of which were previously undocumented. These two novel motifs, motif-7 and motif-6, were identified in the N-terminus of the presumed BCL orthologs. Examination of the composition of these motifs revealed that BCL-motif-6 included the Ala227 residue in its consensus sequence. A recent report showed that the mutated Ala227Gly in the benzoate CoA ligase (BadA) of *R. palustris*, which was designed to reduce the steric clash encountered by substituents during substrate binding and rotation to form benzoate AMP within BadA, increased the activity of BadA toward ortho-substituted substrates designed to bind and rotate within a more sterically favorable active site (Thornburg et al., 2015). In addition, this mutant exhibited low but novel activity toward a meta-substituted benzoate substrate, suggesting the role of BCL-motif-6 in substrate recognition. With respect to BCL-motif-7, we did not find any mutants or experimental procedures indicating its function. Therefore, similar studies should be performed to understand the function of these residues. BCL-motifs-6 and -7 surround BCL-motif-1 in the primary sequence. BCL-motif-1 contains the P-loop that regulates the interaction and binding of phosphate with GDP. The N-terminal domain is reported to contain almost all the residues that bind the carboxylate substrate and the adenosyl group of ATP. In contrast, the C-terminal domain residues coordinate ribose and phosphate groups (Thornburg et al., 2015). Thornburg et al. (2015) study also showed that Lys427, which is fully conserved in our BCL-motif-3, is required for the thioserification reaction. This was also observed by cocrystallization with benzoate and ATP. As mentioned above, the remaining motifs exhibit functional activity in terms of substrate recognition and conversion.

The incorporation of these motifs strongly improved the recognition of BCLs from other aryl-CoA ligases. However, a small group of genomes encode paralogs of the BCL, some of which presented an incomplete genomic context (IC_FM) or a duplication of one motif in the N-terminus (FC_DM). In particular, those with an incomplete context were not assigned initially as orthologs since they lacked one or more subunits of an enzyme, which indicates that the lower degradation pathway of benzoyl-CoA should not be functional. Nevertheless, both preserved a paralogous copy of the transcription factor BzdR/BadR in its genomic context, which may ensure their transcription. The reason why certain bacteria preserve these functional copies has not been determined. Some hypotheses suggest that these redundant pathways may reflect a biological strategy to increase cell fitness in organisms to survive in environments subject to changing oxygen concentrations (Valderrama et al., 2012).

An alternative hypothesis proposes that preserving paralogous copies may function as a bacterial strategy, allowing for variable enzyme concentrations during different growth phases. This phenomenon was also detected in *P. xenovorans* LB400, where proteomic analysis revealed that Box proteins are abundant on the chromosome during the growth phase when biphenyl and benzoate are utilized as carbon sources. Simultaneously, Box proteins in the megaplasmid were detected only in the presence of benzoate during the transition to the stationary phase (Denef et al., 2005). Another study hypothesized that the catalytic efficiency of BCLs would differ for specific substrates (Bains and Boulanger, 2007). These results were inconsistent with those of a proteomic study in which the BCL in the megaplasmid was 60% more efficient at degrading benzoate than was the BCL in the chromosome (Bains and Boulanger, 2007). The authors argue that a possible explanation for the elevated catalytic activity of BCL in the megaplasmid may be that the transcription/translation machinery is probably less active and requires a more efficient enzyme to metabolize the same level of aromatic substrate. However, none of the presented hypotheses fully address why some organisms have two copies of these enzymes. The analysis performed in this work revealed that the copy in the chromosome of *P. xenovorans* LB400 belongs to the IC_FM group, in which the PO of the BoxA subunit is absent, suggesting an impediment for subsequent degradation via this pathway. Considering this result, we hypothesize that the absence of BoxA in the chromosome may impose selective pressure on the copy encoded in the megaplasmid, which possesses a complete Box cluster capable of metabolizing benzoyl-CoA. In this way, the copies in the megaplasmid could complement the absence of BoxA in the chromosome. A complete Box cluster near the BCL could also explain why the megaplasmid enzyme is more efficient but less abundant than the BCL in the chromosome, considering that the next steps in the reaction can be co-transcribed, even for this pathway in which *boxABC* and *boxR* are organized divergently concerning *boxD* and the BCL, as we observed in the genomic context. It has been suggested that increased RNAP binding in one orientation would reduce transcription in the opposite direction in promoter sequences with a divergent organization (Warman et al., 2021). This genomic organization may suggest that the *boxABC* will be more efficiently transcribed in specific conditions than *boxD*, leaving more free enzymes to complement the pathway encoded in the chromosome.

Our data also showed that three *Cupriavidus* species exhibited a BCL in the chromosome where the lower pathway was conspicuously absent. This finding suggested that effective substrate degradation through this pathway requires the expression of Box products encoded by the megaplasmid. Like that of *P. xenovorans* LB400, the chromosomal organization of the BCLs in separate units also indicates that the efficiency of substrate degradation is significantly influenced by the affinity of each enzyme and its concentration.

The constructed phylogenetic tree suggested that species harboring paralogs in multipartite genomes organize each duplicate into distinct clades. Copies positioned closer to the root are encoded on the primary chromosome, indicating their ancestral acquisition compared to those encoded on accessory chromosomes, where some are identified as chromids, as reported in the genera *Cupriavidus* and *Ralsatonia* (Pérez-Pantoja et al., 2012; DiCenzo and Finan, 2017; Dicenzo et al., 2019). The prevailing theory explaining the origin of chromids, known as the plasmid hypothesis, suggests that the inception of the second chromosome involved the acquisition of a megaplasmid through horizontal gene transfer. This event likely occurred early in their evolution and was potentially stabilized by the presence of core genes (diCenzo and Finan, 2017; diCenzo et al., 2019). This hypothesis aligns with the observed differences in the %GC within BCL paralogs, where genes in the chromid predominantly fall within the interquartile range, such as those encoded in the chromosome. Nevertheless, variations in sequence composition persist, placing BCLs in chromids at the boundaries of the interquartile space and those encoded in the chromosome closer to the median. This finding suggested ancestral adaptation of the BCL sequences in chromids to the composition of the genome core.

Some species from the *Azoarcus* and *Thauera* genera also presented paralogous copies of BCL, but both are encoded on the main chromosome. One of the BCLs is associated with a putative anaerobic lower pathway, and the second offers a putative aerobic lower pathway. Notably, the paralogs presented a copy of the transcriptional regulator BoxR and BzdR in their context. As in the case of the species of the family Burkholderiales harboring paralogs of the BCL and the transcriptional regulator, there is no clear answer to why these copies are preserved. A study by Valderrama et al. (2012) showed that in *Azoarcus* sp. CIB, the redundancy of a transcriptional regulator enhances cell fitness by preventing the constitutive expression of *box*/*bad* genes in a benzoate-lacking medium. The authors proposed that the presence of both regulators offers an adaptive advantage, particularly in situations where the functionality of the other regulator offsets the failure of one regulator. However, importantly, the presence of the *boxR* gene in certain bacteria lacking a *box* cluster, such as *R. palustris* CGA009 (Larimer et al., 2004), does not align with its proximity to the anaerobic cluster, as indicated by our results (Supplementary Table S4). Consequently, it cannot be excluded that this regulator may also oversee additional cellular functions that potentially influence bacterial fitness. As evident from various studies and the results presented in this work, the breakdown of benzoate and other monoaromatic hydrocarbons appears to be supported by a substantial level of genetic redundancy. This redundancy likely allows bacteria to exhibit varying degrees of metabolic responses and alternative regulation, enabling them to adapt to challenging environmental conditions.

Our investigation revealed that 87% of the analyzed BCLs and their lower pathways were encoded as single copies in their respective genomes. This phenomenon prompts certain bacteria to maintain only one copy for degrading benzoate and related compounds under conditions of low oxygen levels or depletion. Our findings highlight the broader prevalence of the Box cluster, prompting consideration of the redundancy observed in the aerobic degradation of monoaromatic hydrocarbons within the bacteria under study. This consideration stems from the observed distribution of the classical aerobic benzoate degradation pathway in bacteria, which relies on the hydroxylation of the aromatic ring to produce catechol, which is subsequently cleaved by a dioxygenase, as documented in various studies (Pérez-Pantoja et al., 2003, 2012; Lykidis et al., 2010). The presence of genes coding for both aerobic and anaerobic pathways was confirmed in seven strains belonging to Burkholderiales, including *P. xenovorans* LB400. Notably, the benzoate/catechol and Box pathways of these strains exhibit differential expression under diverse physiological conditions, such as during the growth phase (Denef et al., 2006).

Our research, combined with the findings from Pérez-Pantoja et al. (2012), illustrates the presence of redundancy in Burkholderiales strains. As a result, redundancy within peripheral pathways emerges as a pivotal factor in bacteria engaged in the degradation of monoaromatic hydrocarbons under both aerobic and anaerobic conditions (Pérez-Pantoja et al., 2017). This redundancy significantly influences diverse aspects of bacterial adaptation and metabolism, including growth, detoxification, fitness, and the ability of these versatile bacteria to flourish in environments featuring a variety of monoaromatic carbon sources.

## Conclusion

This research demonstrated that accurately identifying orthologs involves several filtering steps, necessitating features such as conserving protein domains and using the genomic context. These recognition steps were complemented by identifying motifs with structural and catalytic properties, some of them exclusive to BCLs, thereby improving ortholog identification precision and facilitating the recognition of lower degradation pathways as crucial markers for delineating aerobic or anaerobic degradation of benzoyl-CoA. Additionally, the analysis of our predicted distribution confirmed and expanded the functional redundancy of these enzymes through the presence of paralogous copies in certain Betaproteobacteria. Moreover, single-copy pathways complementing aerobic functions with different oxygen requirements were identified. The phylogenetic analysis of BCLs, coupled with phyletic profiles, led to the proposal of a new hypothesis seeking to explain the origin of these paralogs while shedding light on why these bacteria preserved these redundant functions.

With the continuous expansion of available biological data, the bioinformatics community has a unique opportunity to uncover hidden information within sequenced genomes. By utilizing the knowledge generated by our study and employing the proposed techniques, we can significantly enhance the recognition of gene functions in genomes, metagenomes, and genomes assembled from metagenomes collected from distinct environments.

## Data availability statement

The original contributions presented in the study are included in the article/Supplementary material, further inquiries can be directed to the corresponding author.

## Author contributions

CG-P: Data curation, Investigation, Methodology, Software, Validation, Visualization, Writing – review & editing. AL: Investigation, Methodology, Software, Validation, Visualization, Writing – review & editing, Formal analysis. JH: Investigation, Methodology, Software, Validation, Writing – review & editing. R-MG-R: Investigation, Methodology, Software, Validation, Writing – review & editing, Conceptualization, Data curation, Formal analysis, Funding acquisition, Project administration, Resources, Supervision, Visualization, Writing – original draft.

## Supplementary material

Supplementary Table S1 Curated characteristics identified in bacteria featuring experimentally characterized benzoate CoA ligases. The table displays various characteristics sourced from the KEGG database, delineating experimentally characterized benzoate CoA ligases (BCL) and their associated lower pathways in the bacteria *Thauera aromatica*, *Paraburkholderia xenovorans* LB400, *Rhodopseudomonas palustris* CGA009, and *Azoarcus* sp. CIB. These features encompass the genome-gene KEGG identifier, protein name, EC numbers of the BCLs, and the enzymes found in the lower pathway. The table also includes the protein architectures used for predicting orthologs, and the KEGG pathway map, where the BCL and the lower pathway were located.

Supplementary Table S2 Nonredundant sequences. The table displays the amino acid sequences CDHIT chose for predicting ungapped motifs.

Supplementary Table S3 Benozoate CoA ligase homologs. The table presents 234 homologs of the BCL, along with their taxonomy categorized at the class, genus, and species levels. These homologs exhibit the protein architectures of AMP_binding and AMP_binding_C.

Supplementary Table S4 Arrangement of the predicted BCLs within the prediction categories. The predicted BCLs were categorized into FC_FM, IC_FM, and FC_DM, considering the adherence to the proposed recognition steps. The table provides details on the phyletic profile found in the genomic context, taxonomic classification, and predicted catabolism (aerobic, anaerobic, or hybrid) based on the observed phyletic profile for each BCL. The final column specifies the isolation site of the bacterium.

Supplementary Table S5 Benozate CoA ligases which presented the calculated ungapped motifs. The table shows a list of genes for which the motifs describing BCLs were found.
